# Improved site-specific mutagenesis in *Rhodococcus opacus* using a novel conditional suicide plasmid

**DOI:** 10.1007/s00253-022-12204-6

**Published:** 2022-10-04

**Authors:** Garima Jain, Helga Ertesvåg

**Affiliations:** Department of Biotechnology and Food Science, NTNU - Norwegian University of Science and Technology, 7491 Trondheim, NO Norway

**Keywords:** Mycolic acid, Homologous recombination, Conjugative conditional suicide plasmid, *Rhodococcus opacus*

## Abstract

**Abstract:**

*Rhodococcus opacus* PD630 is a biotechnologically important bacterium with metabolic capability for bioremediation, metal recovery, and storage of triacylglycerols. Genome editing by homologous recombination in *R. opacus* is hampered by a very low combined frequency of DNA transfer and recombination. To improve recombination in the species, a conjugative, conditional suicide plasmid based on the replicon derived from the *Corynebacterium glutamicum* plasmid pGA1 was constructed and evaluated in *R. opacus*. The replication of this plasmid is controlled by a dual inducible and repressible promoter system originally developed for *Mycobacterium* spp. Next, we demonstrated that a derivative of this plasmid containing *sacB* as a counterselection marker and homologous regions of *R. opacus* could be used for homologous recombination, and that the problem of obtaining recombinants had been solved. Like for other *Corynebacteriales*, the cell wall of *Rhodococcus* spp. contains mycolic acids which form a hydrophobic and impermeable outer layer. Mycolic acids are essential for *Mycobacterium smegmatis*, but not for *Corynebacterium glutamicum*, and the new vector was used to study if mycolic acid is essential for *R. opacus*. We found that *accD3* that is necessary for mycolic acid synthesis could only be deleted from the chromosome in strains containing a plasmid-encoded copy of *accD3.* This indicates that mycolic acid is important for *R. opacus* viability. The conditional suicide vector should be useful for homologous recombination or for delivering gene products like recombinases or Cas proteins and gRNA to *Rhodococcus* and related genera, while the approach should be applicable for any plasmid needing a plasmid-encoded protein for replication.

**Key points:**

• *Improved vector for homologous recombination in R. opacus*.

• *Mycolic acid is important for survival of R. opacus like it is for Mycobacterium*.

• *Similar conditional suicide plasmids may be constructed for other bacteria*.

**Graphical abstract:**

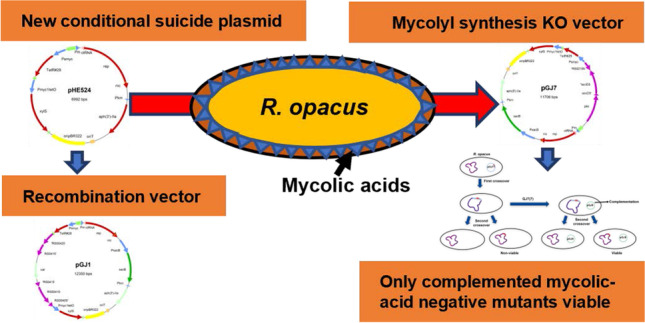

**Supplementary Information:**

The online version contains supplementary material available at 10.1007/s00253-022-12204-6.

## Introduction

*Rhodococcus opacus* PD630 is a Gram-positive, oleaginous actinobacterium that mainly occurs in soil. It stores surplus carbon as triacylglycerols under nitrogen-limiting conditions (Alvarez et al. 2000). *R. opacus* has recently attracted great interest because of its potential industrial applications such as production of large quantities of lipids, high value products from lignocellulosic biomass, production of carotenoids, tolerance towards the aromatic compounds that are generally produced from lignin, and bioremediation by breaking down different organic compounds (DeLorenzo et al. 2018; Anthony et al. 2019; Cappelletti et al. 2020).

Methods for homologous recombination in *R. opacus* are well described, but we found the combined frequencies of plasmid transfer and recombination to be rather low, necessitating several parallel conjugations in order to obtain the desired strains. In this study, we aimed to overcome this bottleneck by developing a conjugative conditional suicide plasmid where the replication in *R. opacus* is dependent on an externally added inducer. When a conditional suicide plasmid is applied for recombination, conjugation can be performed using conditions where the plasmid replicates. Then, recombinants can be selected for by cultivating a transconjugant under conditions where the plasmid does not replicate while simultaneously selecting for a positive selection marker encoded by the plasmid. Our hypothesis was that if the expression of a protein necessary for replication of a plasmid could be controlled with the lowest expression level being below that needed for each daughter cell to always receive a copy of the plasmid, then a conditional suicide plasmid could be constructed. Earlier, similar approaches have been developed and tested in Gram-negative bacteria, achieving a higher success rate for mutagenesis by homologous recombination when gene transfer and recombination were separated in time (Karunakaran et al. 1999; Gimmestad et al. 2009; Naorem et al. 2018).

The order *Corynebacteriales* to which *R. opacus* belongs is known for a unique cell wall structure containing mycolic acids as the major lipid species (Lechevalier et al. 1971; Goodfellow et al. 1982). Mycolic acids are α-branched β-hydroxy fatty acids containing a common mycolic acid motif (2-alkyl 3-hydroxy). The length varies between different species and can be up to 100 carbon atoms long (Marrakchi et al. 2014). The mycolyl-arabinogalactan-peptidoglycan complex forms a unique impermeable and hydrophobic outer layer (Minnikin 1991; de Carvalho et al. 2016; Houssin et al. 2020), which appears to be analogous to the outer membrane of Gram-negative bacteria.

Mycolic acid is formed when two molecules are condensed by the protein Pks13. One of these is a long fatty acyl CoA carboxylated by a specific acyl-CoA carboxylase encoded by *accD3* in *Corynebacterium glutamicum*. The other is the long meromycolic acid which is AMP-activated by Fad32 (Marrakchi et al. 2014). In *R. opacus*, ten genes form the mycolic acid biosynthesis gene cluster (Supplemental Fig. S1); the last of these are three genes homologous to *fad32*, *pks13*, and *accD3* from *C. glutamicum* (Gande et al. 2007). Portevin et al. (2004, 2005) have constructed three different *C. glutamicum* mutant strains that do not produce mycolic acid by deleting *fad32*, *pks13*, and *accD3,* respectively, although the mutants grew slower than the wild type. However, the corresponding genes could not be deleted in *Mycobacterium smegmatis*, suggesting that mycolic acids are essential for *Mycobacterium* but not for *Corynebacterium* (Portevin et al. 2004, 2005).

The objectives of this study were twofold. Firstly, we addressed the bottleneck for mutagenesis by homologous recombination by constructing a conditional suicide plasmid based on a cryptic plasmid pGA1 found in *C. glutamicum* (Nesvera et al. 1997). pGA1 encodes the Rep protein that is needed for replication of the plasmid. The amount of Rep and hence the plasmid’s copy number is controlled by a ctRNA overlapping the start of *rep* (Tauch et al. 2002). Several promoters were tested in this study, and the most promising was used to replace the endogenous promoter for *rep*. Secondly, the studies mentioned above on mycolic acid mutants addressed *Mycobacterium* and *Corynebacterium*, but not other genera in the order. It has been shown that the long mycolic acids are not necessary for growth of *Rhodococcus equii* and that growth of this bacterium was much less inhibited by the FASII inhibitors triclosan and isoniazid compared to *M. smegmatis* (Sydor et al. 2013). Still, this only indicates that the two genera might be different, while it still is not known if mycolic acid biosynthesis is necessary for *R. opacus*. Hence, we chose to test our new vector by trying to inactivate *fad32*, *pks13*, and *accD3*, using a similar approach as the one utilized by Portevin et al. (2004, 2005).

## Methods

### Strains, growth media, and culture conditions-

*R. opacus* PD630 (DSM 44193 renamed *Rhodococcus wratislaviensis*) was cultured at 30 °C and *Escherichia coli* at 37 °C, both at 225 rpm shaking in liquid Luria Bertani (LB) media (10 g/L tryptone, 5 g/L yeast extract, 5 g/L NaCl) or on LA plates (LB containing 15 g/L agar). Six percent sucrose was added to the solid media when needed for selection against SacB. For selection of transformants and conjugants, the concentrations of antibiotics used were kanamycin (Km) 25 μg/mL, chloramphenicol (Cm) 10 μg/mL, nalidixic acid (Nal) 40 μg/mL, and gentamycin (Gm) 20 μg/mL for *R. opacus* and Km 50 μg/mL, Gm 20 μg/mL, and Cm 20 μg/mL for *E. coli*. Gene expression was induced by 0.5 mM m-toluate or repressed by 100 ng/mL anhydrotetracycline (aTc) unless stated otherwise. *E. coli* DH5α was used for cloning and *E. coli* S17.1 (Simon et al. 1983) for conjugation*.*

### Construction of plasmids

All plasmids and the details of their construction are described in Supplementary Table S1. All primers were designed using the Clone Manager 9 software from Sci Ed Software LLC, USA, and are listed in Supplementary Table S2. Plasmids constructed with PCR-amplified fragments were verified by Sanger sequencing (Eurofins Scientific, Luxembourg). Restriction enzymes, DNA modification enzymes, T4 DNA ligase, and Q5® High-Fidelity DNA Polymerase were obtained from New England Biolabs, USA. Zero Blunt™ TOPO™ PCR Cloning Kit (Invitrogen, USA) was used for cloning of some of the PCR fragments. SLIC cloning was performed as described earlier (Islam et al. 2017). For genomic DNA extraction, MasterPure™ Complete DNA and RNA Purification Kit (Lucigen, USA) was used, but cells from one 1 ml culture were treated with lysozyme (0.5 mg/ml) in 150 µl TE for 30 min prior to treatment with proteinase K. All construction of plasmids was performed using *E. coli*, and the final plasmids were then transferred to *R. opacus* by conjugation.

### Construction of strains by conjugation and homologous recombination in *R. opacus*

Conjugation was performed as described earlier (Gimmestad et al. 2009), and the plasmid-encoded kanamycin or gentamycin resistances were used to select against *R. opacus* wild-type cells, while nalidixic acid was used to select against *E. coli.* For recombination studies using conditional suicide plasmids, the first crossover was selected for by omitting the inducer (m-toluate) and adding the repressor (aTc) to the medium. Double crossovers were selected on LA sucrose plates, and sucrose-resistant colonies were checked for sensitivity to kanamycin and further tested by PCR to identify clones with the desired mutant genotype. For colony PCR, some cell mass was transferred to the 1.5-mL tubes with 100 μL milli-Q water and pipetted up and down to mix the solution. The cells were then incubated at 100 °C for 15 min, and 0.5–1 µl of the boiled solution was used as DNA sample for PCR reactions. When necessary, isolated genomic DNA was used instead of boiled cells.

### Assay of promoter strength

The Luciferase Assay System E1500 (Promega corporation, USA) was used to quantify the production of luciferase. Strains with plasmids were inoculated from precultures to an initial OD600 of 0.05, cultivated in LB Km and sampled after 24 and 48 h. *R. opacus* (pEC18Kmob2) was used as negative control for luciferase. All cultivations were performed in triplicates.

## Results

### Comparison of different promoters

**Fig. 1:**
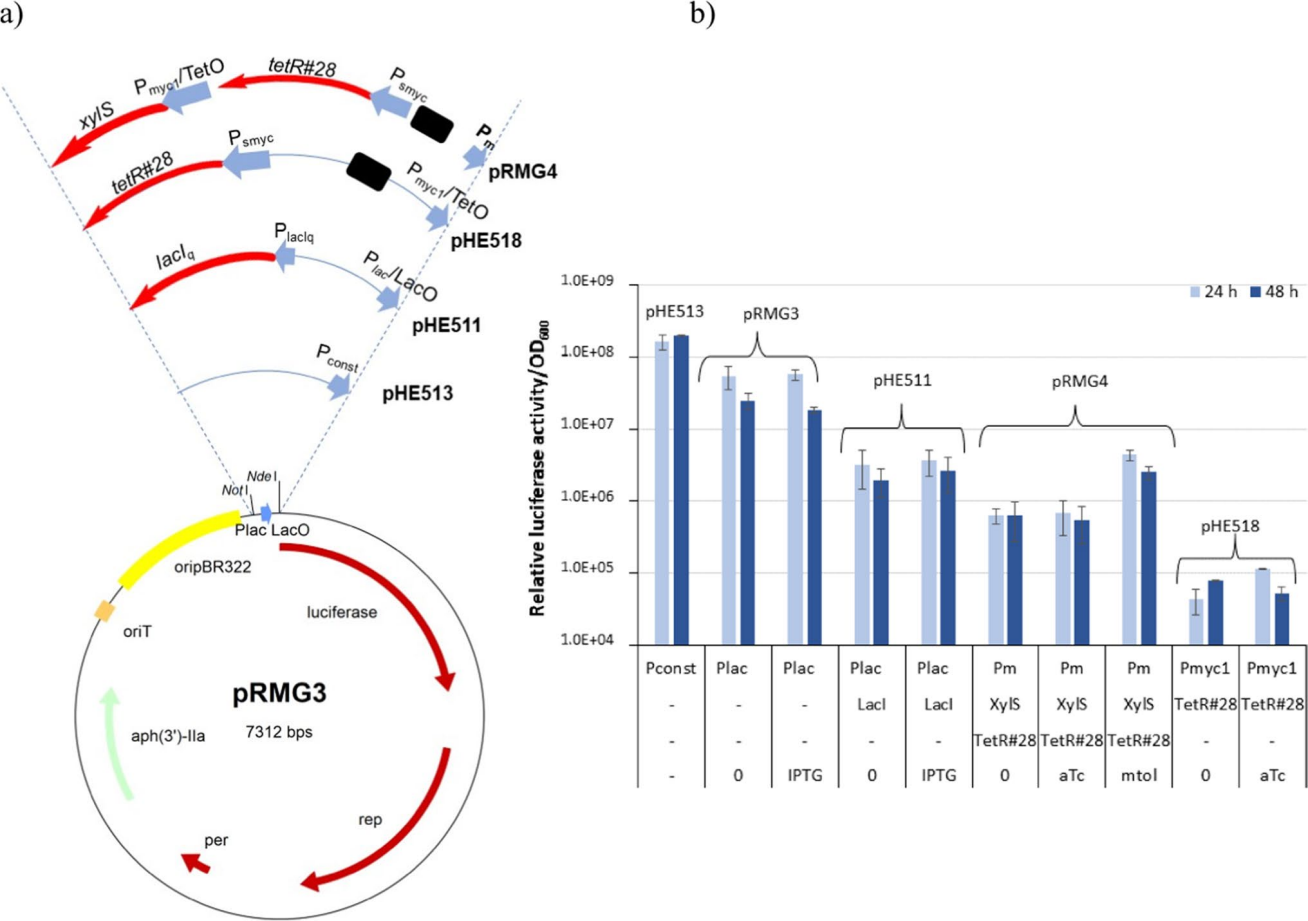
**a** Map of the plasmids used to test the expression systems. The *Not*I and *Nde*I restriction sites were used to exchange promoters and control elements. Promoters and operators are shown as thick arrows; the black boxes indicate the rrnBT1T2 transcriptional terminator. The intervening DNA is not shown in scale. **b** Promoter strength in *R. opacus* measured as luminescence/OD_600_. *R. opacus* cells containing these plasmids (label above bars) were cultivated in LB containing Km, and samples were collected after 24 and 48 h. The numbers are given as the average of three biological replicates. The first line in the horizontal axis shows the promoter used to express the luciferase gene, the second the regulatory protein, the third the regulatory protein used to regulate expression of the first regulatory protein, and the last line the addition of repressor or inducer. “–” is used when regulatory proteins or repressors/inducers are not relevant for that plasmid

Our first task was to find a promoter system for the *rep* gene that preferentially could be turned on and off, and with sufficient promoter strength to allow for good plasmid replication when the promoter was turned on. In order to compare different promoter systems, it is important that the vectors are as identical as possible. A vector backbone based on pEC18Kmob2 (Tauch et al. 2002) was constructed and denoted pRMG3 (Fig. 1a). pRMG3 was constructed by adding a *Nde*I-site downstream of the P_*lac*_ and RBS found in the vector, cloning a luciferase-encoding open reading frame from the plasmid pUV15tetORm::luciferase into this *Nde*I-site, and finally add a *Not*I site upstream of the P_*lac*_ promoter. The promoter is then flanked by *Not*I and *Nde*I restriction sites, and these were used to exchange this promoter with other promoters and control elements as depicted in Fig. 1a. Co-expression of *lacI* together with the P_*lac*_-promoter (pHE511) was tested since we were looking for an inducible system. In pHE518, production of luciferase was controlled by the strong *P*_*myc*1_ promoter from *M. smegmatis* combined with the tet-operator TetO (Ehrt et al. 2005). The TetR variant used, TetR#28, functions as a repressor for P_*myc1*_ with anhydrotetracycline as corepressor (Klotzsche et al. 2009). We also tested a double control system developed for *Mycobacterium tuberculosis* (Dragset et al. 2015) in which the TetR#28 repressor variant is used to repress expression of *xylS* from the *P*_*myc*1_/TetO promoter/operator. In this plasmid (pRMG4), the luciferase gene is expressed from the *P*_*m*_ promoter, which depends on XylS together with its coactivator m-toluate for efficient expression. For comparison, we included the constitutive promoter P_*const*_ (pHE513) that has been analyzed earlier (DeLorenzo et al. 2018).

The plasmids were transferred to *R. opacus* by conjugation, and the strains were cultivated in LB containing Km and the appropriate inducers and/or repressors, and growth and luciferase activity were measured. Measured luciferase activity per OD_600_ was then calculated and used to evaluate relative promoter strength (Fig. 1b). The results showed a difference of 1000 × in luciferase activity per cell. P_*const*_ was the strongest of the tested promoters, followed by P_*lac*_ while P_*myc1*_ was the weakest. Induction by IPTG or repression by aTc did not have any significant effect on P_*lac*_ or P_*myc1*_, respectively. Still, co-expression of LacI resulted in a tenfold reduction in luciferase activity as compared to the cells containing the smaller plasmid pRMG3. In the absence of m-toluate, the luciferase activity in cells containing pRMG4 with the P_*m*_ promoter was five times less than for cells containing *lacI* and P_*lac*_ (pHE511). However, when the inducer m-toluate was added, the promoter strength of the P_*m*_-construct was similar to that of LacI-P_*lac*_ (Fig. 1b). We did not observe any effect of using aTc in liquid media, but higher amounts of the repressor could have been tested.

### Construction and verification of a conditional suicide plasmid

pEC18Kmob2 is based on plasmid pGA1 and encodes the Rep protein necessary for its own replication (Tauch et al. 2002). The plasmid replicates by a rolling circle mechanism, and the replication site, denoted “*nic*,” is found at the 3′-part of *rep* (Abrhámová et al. 2002). Since only the construct with a double control (P_*myc1*_/TetO and P_*m*_) displayed inducible luciferase activity, we chose to use that system to control the protein necessary for replication in plasmid pEC18Kmob2. It should be noted that we did not know if the induced promoter strength would be sufficient for replication, or if the uninduced expression level was sufficiently low to make the plasmid unstable. However, this regulated promoter system seemed to be the most promising of those tested. The expression of *rep* is also controlled by a ctRNA overlapping the start codon and promoter of the gene (Venkova-Canova et al. 2003). This could be important for the stability of the plasmid, and two different versions were constructed: pHE524 containing the ctRNA and pHE523 containing only the ORF of *rep.*

**Fig. 2:**
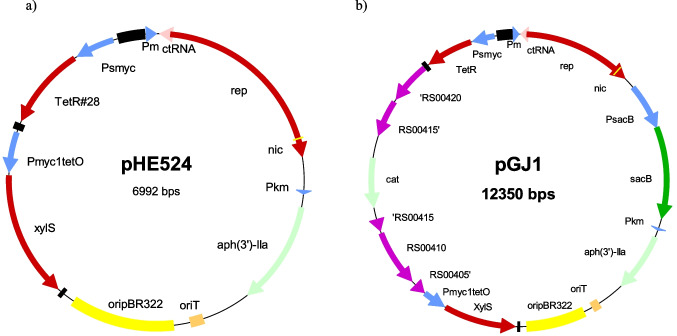
Maps of the **a** conjugative, conditional suicide plasmid pHE524. The elements used to control replication of the plasmid in *R. opacus* (*nic, rep*, TetR#28, and XylS) are described in the text. oripBR322 is used for replication in *E. coli* and oriT is necessary for conjugative transfer of the plasmids. *aph*(3,)-IIa encode resistance to kanamycin. **b** The pHE524 derivative pGJ1 used for homologous recombination in *R. opacus*, displaying the insertion of homologous arms flanking the chloramphenicol resistance gene *cat* and the SacB-encoding cassette used for negative selection of the second recombination step. Terminators are indicated as black boxes

The plasmids were transferred to *R. opacus* by conjugation, and many transconjugants were obtained for both plasmids when selected for on plates containing Km, Nal, and m-toluate. Transconjugants were then cultivated in LB with and without aTc and plated on LA Km with and without aTc, and on LA as a control for CFU. The results (Table 1) show that even without aTc in the medium, the plasmids were lost at a high frequency. There was no strong effect of the added aTc. Moreover, the cells did not grow well on plates with both Km and aTc, indicating some effect of the repressor on plasmid replication on the solid medium. The plasmid containing ctRNA, pHE524 (Fig. 2a), was lost ten times more frequently than the one without this element (Table 1) and was chosen as the best conditional suicide vector.

**Table 1:** The stability of the plasmids with and without ctRNA. Transconjugants were cultivated in LB with and without aTc and were then plated on LA with and without Km and aTc

		Growth on different solid media (CFU/ml)			
	Cultivated in	LA	LA Km	LA Km aTc	Frequency of retaining the plasmid *
pHE523 no ctRNA	LB	6.50E + 08	1.40E + 06	Tiny	2.15E − 03
	LB aTc	1.06E + 09	1.10E + 06	Tiny	1.04E − 03
pHE524 with ctRNA	LB	5.60E + 08	1.09E + 05	Tiny	1.95E − 04
	LB aTc	1.03E + 09	2.60E + 05	Tiny	2.52E − 04

^*^Calculated as CFU (LA Km)/CFU (LA)

### The constructed conditional suicide plasmid can be used for homologous recombination

The next step would then be to demonstrate that this conditional suicide plasmid can be utilized for homologous recombination in *R. opacus.* As a first and simple test for the plasmid, we chose to inactivate gene *PD630_RS00415*, one of eleven genes in the genome that putatively encode fatty acyl CoA ligases. We have previously inactivated this gene in *R. opacus* using a conventional, non-replicative recombination plasmid, pMV11 (Supplemental Fig. S2), where *PD630_RS00415* was inactivated by an inserted Cm^r^-gene and knew that this mutant grew similar to the wild-type strain (unpublished). The same inactivated copy of *PD630_RS00415* as in pMV11 and the *sacB* gene encoding levan sucrase to enable selection of the second recombination step were inserted into pHE524 creating pGJ1 (Fig. 2b). The two plasmids were then conjugated to *R. opacus.* For each plasmid, three parallel conjugations were performed, and transconjugants were selected on plates containing Km for pGJ1 and Gm for pMV11; in both cases, the plates also contained Nal to select against *E. coli*. No transconjugants were found after conjugation using pMV11; we usually expect 0.1 to 0.2 transconjugants per experiment. However, transfer of pGJ1 resulted in several hundred transconjugants on selective medium and a conjugation frequency of around 3 per 10^6^ recipient cells (Supplemental Fig. S3). We also tested the potential effects of enhancing replication by adding the inducer m-toluate to the conjugation plates and the effect of adding the replication inhibitor aTc to the selection plates. However, neither of these treatments resulted in any significant change in the conjugation frequency.

**Fig. 3:**
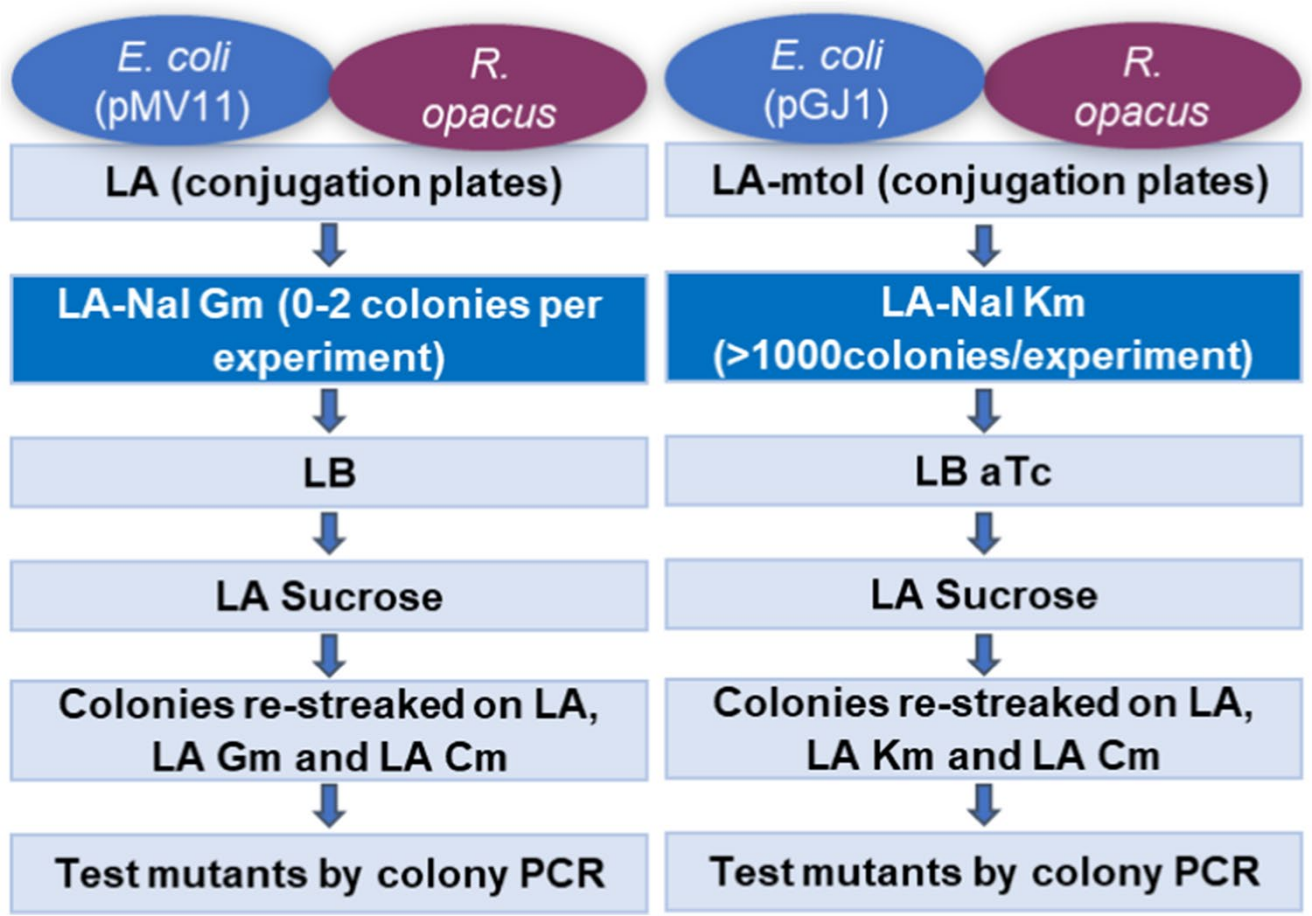
Conjugation scheme using the traditional suicide plasmid (example pMV11) and the conditional suicide plasmid (example pGJ1). The improved step is emphasized

When a plasmid is used to transfer the homologous fragments flanking changed DNA, a double crossover is needed to obtain the mutant, and this necessitates a multi-step selection protocol (Fig. 3). In the first step, the plasmid will integrate through recombination with one of the homologous regions. In the second step, a second recombination event will result in either wild-type or mutant strains. After transferring the plasmid through conjugation in *R. opacus*, three transconjugant colonies were picked from a Nal, Km plate, and each was cultivated in LB containing Km, LB with aTc, LB with m-toluate, and LB with no additive. Dilutions were plated from each culture on LA, LA Km, and LA sucrose plates. After growth in liquid media without kanamycin, about 1% of the cells grew on LA Km. This is a relatively high number compared to those found for pHE524 (Table 1) indicating that some recombination had taken place at a previous step. Growth on sucrose plates could be caused by plasmid loss or by a successful double recombination, although it is also known that some cells might grow on sucrose despite the presence of *sacB* (Hashimoto et al. 2003). Therefore, cells from the sucrose plates were restreaked to plates with either Km or Cm since successful double recombinants should be Km^s^ and Cm^r^. Four Cm^r^, Km^s^, sucrose^r^ colonies were confirmed by colony PCR to contain the inserted Cm-gene (Supplemental Fig. S4), demonstrating that the conditional suicide plasmid can be used for homologous recombination in *R. opacus*. The final protocol (Fig. 3) has the same number of steps as the one using a standard suicide plasmid, but the challenge of few or none transconjugants had been overcome by the new conditional suicide plasmid.

### Genes in the mycolic acid gene cluster could not be easily inactivated by homologous recombination

The hydrophobicity of the *R. opacus* cells results in properties like foaming in high-density cultures and adherence to each other and to some particles. Mycolic acids might be important for these properties, and mycolic acid–negative cells could be useful as control strains in some experiments exploring the surface properties of *R. opacus*. The second objective of this study was to see if it is possible to remove the genes necessary for mycolic acid biosynthesis in *R. opacus* like it has been done for *C. glutamicum* (Portevin et al. 2005). Plasmid pGJ6 (Supplemental Fig. S5) is similar to pGJ1, but that the target is the three genes *fad32-pks-accD3* at the 3′ end of the mycolic acid gene cluster (Supplemental Fig. S1). At the end of the recombination protocol (Fig. 3), 17 sucrose^r^, Km^s^ colonies were tested by colony PCR using 6 different primer pairs. We found that although strains where the plasmid was incorporated into the genome had been obtained, only wild-type strains were obtained after the second cross over (Supplemental Fig. S6). The whole experiment was repeated, and this time, 30 sucrose^r^, Km^s^ colonies were tested after genomic DNA extraction, but again, we ended up with the similar negative results (not shown).

These results indicated that mycolic acid could be essential for the survival of *R. opacus* like it is for *M. smegmatis* (Portevin et al. 2004, 2005)*.* In their study, this was demonstrated by showing that deletion of essential genes in mycolic acid biosynthesis in *M. smegmatis* was possible only if a copy of the same gene was expressed from a complementation plasmid (Portevin et al. 2005). Using their approach, a new recombination plasmid, pGJ7 (Supplemental Fig. S5), was constructed targeting *accD3*, the last gene in the operon*.* pGJ7 contains the homologous regions flanking both sides of a 533 nt partial *accD3* deletion (Supplemental Fig. S7). pGJ7 was conjugated to *R. opacus*, and transconjugants were cultivated in the presence of aTc and kanamycin for recombination (Supplemental Fig. S8). We had found that cells with the suicide plasmid did not grow well on LA plates with Km and aTc (Table 1); hence, this step was included to select for the recombinants as depicted in Supplemental Fig S8. After selecting for cells that grew on the LA Km aTc plates (Supplementary Fig S8), 4.26 × 10^6^ colonies/ml culture were obtained. These two extra steps might not have been necessary but were added to ensure that strains with an integrated copy of the plasmid were obtained. Colonies picked from the LA Km aTc plates were shown by colony PCR to be first recombinants. One such strain containing pGJ7 inserted into the genome was named GJ7(7) and chosen for the further studies (Supplemental Fig. S9). Similar to the recombination experiments using pGJ6, after selection on sucrose, only wild-type cells or cells that still retained the integrated plasmid were found (Supplemental Fig. S9). The latter probably resulted from inactivation of *sacB*; this selection system is known to give some false positives (Cianfanelli et al. 2020).

**Fig. 4:**
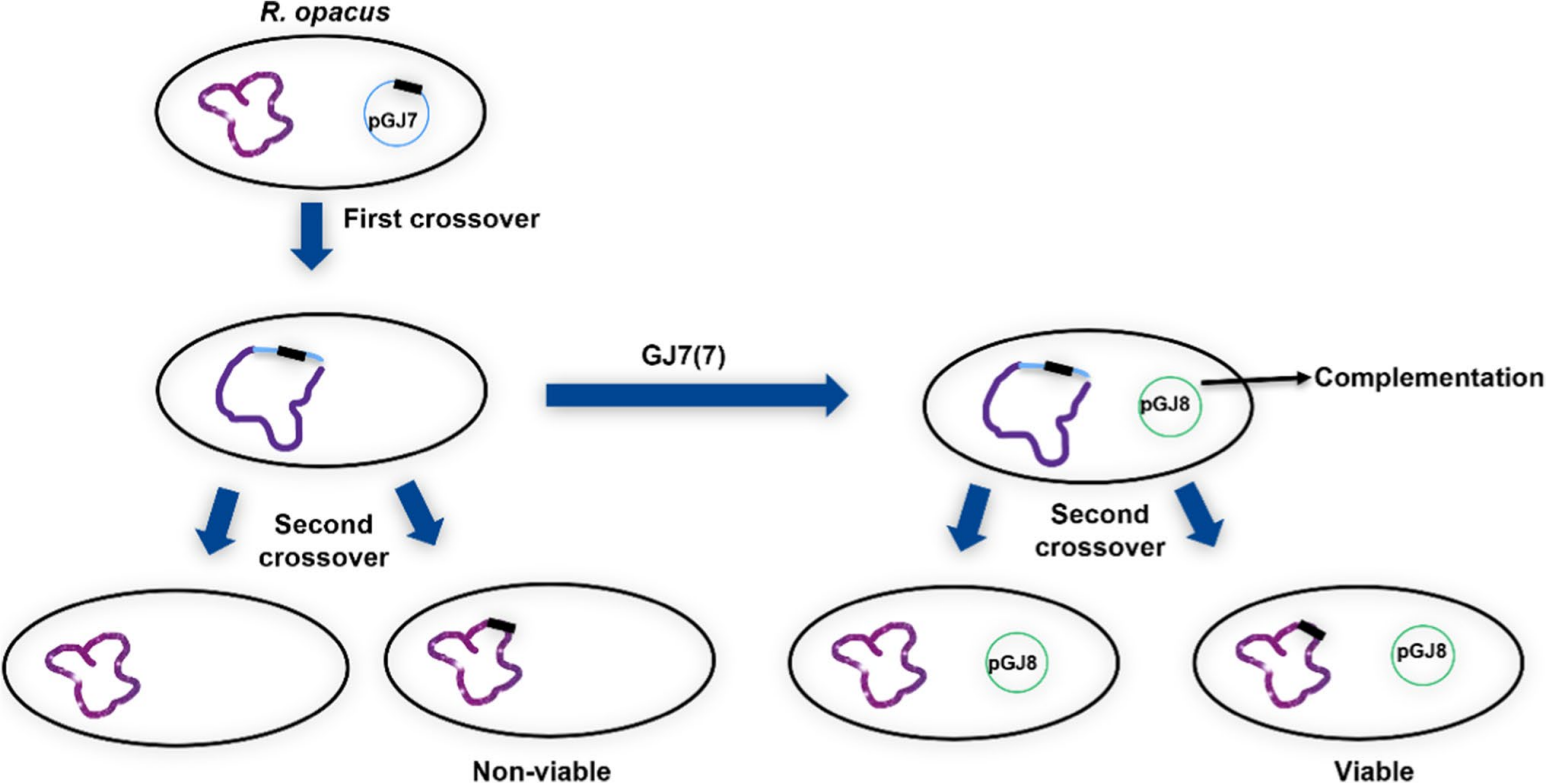
Overview of homologous recombination approach for deletion of *accD3* in *R. opacus* using conditional suicide plasmid pGJ7 and complementation plasmid pGJ8. The gene of interest (*accD3*) with the partial deletion is depicted as a thick, black line

However, first recombinants like strain GJ7(7) could be used to see if *accD3* could be deleted in the chromosome when a plasmid expressing *accD3* was present in the cell. To do so, complementation plasmid pGJ8 was constructed. In this plasmid, *accD3* is controlled by the strong P_*const*_. Plasmid pGJ8 was then transferred to strain GJ7(7) by conjugation and selected for by Gm (Supplemental Fig. S8). Then, double cross-over events were selected for by sucrose, and the strains were subsequently tested for sensitivity to Km. This is the same approach as used by Portevin et al. (2005) and is illustrated in Fig. 4 and Supplemental Fig. S8.

**Fig. 5:**
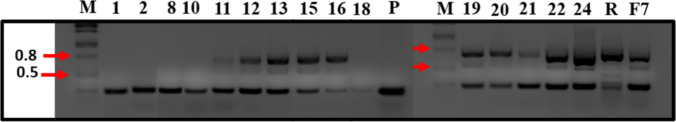
Gel image showing *R. opacus* Δ*accD3* mutant strain resulting in presence of complementation plasmid, tested with primers delaccD3F/R. Expected bands for wild type: 0.719 kb, mutant: 0.192 kb and first recombinants: 12.4, 0.719, and 0.192 kb. R: *R. opacus* (wild type), F7: GJ7(7), P: pGJ7, M: standard (PstI-restricted phage *λ* DNA)

Genomic DNA was extracted from 15 sucrose^r^, Km^s^, and Gm^r^ colonies and tested by PCR. Five of the colonies showed the mutant band (0.192 kb), while ten showed bands corresponding GJ7(7) (0.192 and 0.719 kb) (Fig. 5). Surprisingly, none of the mutants had reverted to the wild type. The PCR products for two of the mutants (#2 and #8) were confirmed by sequencing to rule out the possibility of non-specific PCR products of the apparently correct length. The result strongly indicates that biosynthesis of mycolic acid is important for the viability of *R. opacus.*

## Discussion

*R. opacus* is among those bacteria where the combined rate of conjugation and rate of recombination is rather low. Therefore, it becomes laborious and time consuming to obtain strains where the plasmid has integrated into the chromosome when non-replicating plasmids are used. There is no reason why homologous recombination cannot take place even if a replicating plasmid were used; however, it would then be very difficult to select for the mutants or to get rid of the plasmid after a successful second recombination had removed it from the chromosome. The latter would be necessary to avoid new rounds of recombination events.

In this work, we developed a conditional suicide plasmid for *R. opacus* (Fig. 2)*.* Obviously, a much higher number of transconjugants were obtained when this plasmid was used for homologous recombination compared to using a non-replicating plasmid. Since the process of plasmid transfer became decoupled from that of recombination, the plasmid solved the problem of the low number of transconjugants obtained when using non-replicating recombination plasmids.

The plasmid was then used to delete essential genes in the mycolic acid biosynthetic pathway of *R. opacus.* However, a chromosomal deletion mutant was only obtained in the presence of a complementing plasmid. Earlier, Portevin et al. used a similar approach to show that a mutant with deletions of *pks13*, *fad32*, or *accD4* was viable in *M. smegmatis* only when a complementing plasmid expressing these genes was present. However, in their study, they used a thermosensitive plasmid as their conditional suicide plasmid (Portevin et al. 2004, 2005). Our results suggest that mycolic acid is essential for the viability of *R. opacus* like it is for *Mycobacterium* but not for *Corynebacterium* (Portevin et al. 2004, 2005; Raad et al. 2010). For the latter genus, one species lacking mycolic acid has also been described (Collins et al. 1988).

Measurements of relative promoter strengths (Fig. 1b) combined with the identification of pHE524 as a conditional suicide plasmid (Table 1) indicate that a promoter strength above the uninduced level of our dual control system (pRMG4) but below the induced level is necessary for the plasmid to be stably maintained. If the tested promoters work equally well for the *rep* gene as for the luciferase-expressing gene, it follows that if P_*cons*t_ or P_*lac*_ had been used to control *rep*, the plasmid would have been too stable. If P_*myc1*_ had been used, the plasmid would have been less stable since that promoter seemed weaker than the uninduced P_*m*_ dual control system. Still, it might have been sufficient for recombination purposes, especially if the ctRNA had been removed.

Similar conditional suicide plasmids to ours have earlier been developed for Gram-negative bacteria (Gimmestad et al. 2009; Naorem et al. 2018), and this suggests that the approach may be utilized in other species as well, if an inducible or repressible promoter is available for the species. The P_*m*_-XylS expression system was chosen as one of our candidates because of its known broad host range (Gawin et al. 2017), and our result shows that the system constructed for *M. tuberculosis* (Dragset et al. 2015) provided inducible expression in *R. opacus*, too. Even though we did not observe a significant effect of aTc in liquid culture for *R. opacus*, the system still worked well. It would have been possible to test higher concentrations of the repressor if we had needed the effect. As it is, our results show that when a fairly weak promoter is used to express *xylS*, further repression of this promoter was not necessary for obtaining a suitably unstable delivery plasmid in *R. opacus*. For Gram-negative bacteria, it has been shown that *P*_*m*_ promoter mutants and 5′UTR mutants with a wide range of expression strengths may be found (Bakke et al. 2009; Lale et al. 2011), and this could be an alternative if the wild-type promoter has a too low induced activity or a too high uninduced activity in the bacterium of interest.

The conditional suicide vector pHE524 should also be useful as a vector for delivering, e.g., transposons or genome-editing systems like CRISPR-Cas or Cre-Lox, because the plasmid disappears with high frequency when it is not selected for by kanamycin. Moreover, since the replication system originates from *C. glutamicum* pGA1 (Tauch et al. 2002), while the promoter-regulator system controlling replication in the conditional suicide vector was developed for *M. smegmatis* (Dragset et al. 2015), the plasmid could potentially function in the genera *Mycobacterium* and *Corynebacterium* as well as for *Rhodococcus*.

## Supplementary Information

Below is the link to the electronic supplementary material.

Supplementary file1 (PDF 1.44 MB)

## Data Availability

All data generated or analyzed during this study are included in this published article and its supplementary information files. Plasmids are available upon request.
