# Supplementary material for: Improved site-specific mutagenesis in Rhodococcus opacus using a novel conditional suicide plasmid
Source: Appl Microbiol Biotechnol. 2022 Oct 4;106(21):7129–38. doi: 10.1007/s00253-022-12204-6 (PMC9592669; doi:10.1007/s00253-022-12204-6)
Supplement: Supplementary file 1 — Supplementary file1 (PDF 1.44 MB) [file 253_2022_12204_MOESM1_ESM.pdf]

Supplementary Information

Applied Microbiology and Biotechnology

Improved site-specific mutagenesis in *Rhodococcus opacus* using a novel conditional suicide plasmid

Authors

Garima Jain[1], Helga Ertesvåg[1]*

[1]Department of Biotechnology and Food science, Norwegian University of Science and Technology (NTNU), Trondheim, NO-7491, Norway

*Correspondence to: helga.ertesvag@ntnu.no

Supplementary Table S1: Plasmids used in the study.

Name	Description	Source
pEC18Kmob2	pGA1-based expression vector. P_{lac} , Km^r	(Tauch et al. 2002)
pUV15tetORm:: luciferase	pAL5000 based vector. Luciferase gene *(luc)* controlled by P_{myc1}*tetO*. TetR controlled by P_{imyc}, Km^r.	(Grant et al. 2013)
pMDXluc	pAL5000 based vector, *xylS* controlled by P_{myc1}/TetO, TetR reverse repressor variant #28 (TetR#28) controlled by P_{smyc}, *luc* controlled by *Pm*. Km^r.	(Dragset et al. 2015)
pXMJ19	*E. coli*-*C. glutamicum* shuttle vector. P_{tac}, *lacI*^{q}, Cm^r.	(Jakoby et al. 1999)
pDD120	Derivative of pB264. P_{const}. Km^r	(DeLorenzo et al. 2018)
pCR™Blunt II-TOPO®	ColE1-derivative, *ccd*B, $Zeocin^r$, Km^r.	Life Technologies
pJQ200mp18	P15-based suicide vector. SacB. Gm^r.	(Quandt and Hynes 1993)
pHE95	Conditional suicide plasmid for Gram-negative bacteria. Km^r, Ap^r.	(Gimmestad et al. 2009)
pRMG2	Derivative of pEC18Kmob2 containing *luc* from pUV15tetORm:: controlled by P_{lac}. Km^r	This study
pRMG3	Derivative of pRMG2 with a *Not*I-linker (New English Biolabs Inc) replaced a small *Ase*I-DNA fragment, Km^r	This study
pRMG4	Derivative of pRMG3 where a *Not*I-*Nde*I fragment from pMDX luc containing *Pm* and encoding the regulatory proteins XylS and TetR#28 replaced P_{lac}. Km^r	This study
pRMG5	Derivative of pRMG4 where a *Not*I-*Eco*RV fragment encoding XylS was removed. Km^r	This study
pHE511	A 1.2 kb PCR fragment (primer pair 1/2) from pXMJ19 encoding *lacI*^{q} was inserted (SLIC) into *Not*I-restricted pRMG3. Km^r	This study
pHE513	Derivative of *Sac*I-*Nde*I digested pRMG5 where a *Sac*I-*Nde*I fragment from pDD120 containing P_{const} was inserted. Km^r	This study
pHE518	Derivative of *Bsi*WI-*Nde*I digested pRMG5 where a BsiWI-NdeI fragment from pUV15tetORm::luciferase containing P_{myc1}/*T*etO was inserted. Km^r	This study
pHE523	Two PCR fragments from pRMG4 generated using primer pairs 3/4 and 5/6, were combined. Contains the control elements from pMDXluc including the 5' region between P_m and NdeI, but P_m is controlling *rep*. Km^r	This study

pHE524	Two PCR fragments from pRMG4 generated using primer pairs 4/7 and 6/8, were combined. Contains the control elements from pMDXluc but P_m is controlling *rep.* The UTR of *rep* with the ctRNA is retained. Km^r	This study
pMV10	Derivative of pCR™Blunt II-TOPO® where three PCR products were combined. Contains 1.1 kb including the 5' part of *PD630_RS00415* (primers 9/10) and 1.2 kb including the 3' part of this gene (primers 11/12) separated by *cat* from pXMJ19 (primers 13/14)	This study
pMV11	Derivative of pJQ200mp18 in which a 3.5 DNA fragment from pMV10 was inserted. Gm^r Cm^r. Km^r	This study
pMW3	A 1.2 PCR fragment (primers 15/16) from *R. opacus* containing *accD3* inserted in pCR™Blunt II-TOPO®, Km^r	This study
pMW4	A 4.2 kb PCR fragment (primers 17/18) from *R. opacus* encoding *fad32* and the 5' part of *pks*. Km^r	This study
pDD112	Derived from pNG2, chloramphenicol resistance gene (CM) optimized for *R. opacus*, P_{const}, Gm^r	(DeLorenzo et al. 2018)
pGJ1	Derivative of pHE524 in which two PCR fragments were SLIC cloned, a 3.5 kb PCR fragment (primers 19/20) from pMV10 containing *PD630_RS00415* interrupted with *cat* and a 2kb, PCR fragment encoding *sacB* from pHE95 (primers 33/34), Km^r	This study
pGJ2	Derivative of pCR™Blunt II-TOPO® where a 1 kb PCR fragment (primers 21/22) from pDD112containing CM, P_{const} and *rrnB* was inserted, Km^r, Cm^r	This study
pGJ3	The 1 kb *Xba*I/*Bam*HI digested DNA fragment from pGJ2 containing *cat*, P_{const} and *rrnB* ligated with a 4.8 kb SpeI/BglII digested fragment of pMW4 containing the 5' 1.3 kb of *fad 32*, Km^r, Cm^r	This study
pGJ4	Ligation of a *Bam*HI/*Xba*I digested DNA fragment from pGJ3containing *cat* and *fad32'* and a 1.4 *Bgl*II/*Xba*I digested DNA fragment from pMW3 containing the 3' part of *accD3* and parts of the downstream gene. Km^r, Cm^r	This study
pGJ5	A *Psh*AI/*Xba*I digested DNA fragment encoding *sacB* from pGJ1 was ligated to *Psh*AI/*Xba*I digested fragment of pHE524. Km^r	This study
pGJ6	Derivative of *Bsi*WI cut pGJ5 where a 4.1Kb *Bsr*GI/*Acc*651 restriction fragment from pGJ4 containing ~1 Kb homologous construct flanking each side of CM was inserted. Km^r, Cm^r , Fig. S5	This study
pGJ7A	1.2 kb PCR fragment (primers 23/24) containing 3' part of *pks* (1107 bp) and some 5' part of *accD3* (69 bp) from *R. opacus* total DNA, cloned in pCR™Blunt II-TOPO®, Km^r	This study
pGJ8A	Complete *accD3* excised on 1.56 kb *Nde*I/*Eco*RI restriction fragment from pMW3 and ligated into corresponding sites of pHE513. Km^r	This study
pGJ7	A 1.2 kb *Nsi*I/*Age*I restricted DNA fragment from pGJ7A containing part of *pks* and the 5' part of *accD3* replaced the *fad32-CM* encoding parts of pGJ6. Km^r, Fig. S5	This study
pGJ8	PCR fragment (primers 25/26) from pGJ8A carrying *accD3* and UTR+RBS, SLIC cloned into SphI/DraIII cut pDD112 P_{const}, Gm^r	This study

References to Table S1

DeLorenzo DM, Rottinghaus AG, Henson WR, Moon TS (2018) Molecular toolkit for gene expression control and genome modification in *Rhodococcus opacus* PD630. ACS Synth Biol 7:727-738 doi:10.1021/acssynbio.7b00416

Dragset MS, Barczak AK, Kannan N, Mærk M, Flo TH, Valla S, Rubin EJ, Steigedal M (2015) Benzoic acid-inducible gene expression in *Mycobacteria*. PLoS One 10:e0134544 doi:10.1371/journal.pone.0134544

Gimmestad M, Ertesvåg H, Heggeset TMB, Aarstad O, Svanem BIG, Valla S (2009) Characterization of three new *Azotobacter vinelandii* alginate lyases, one of which is involved in cyst germination. J Bacteriol 191:4845-53

Grant SS, Kawate T, Nag PP, Silvis MR, Gordon K, Stanley SA, Kazyanskaya E, Nietupski R, Golas A, Fitzgerald M, Cho S, Franzblau SG, Hung DT (2013) Identification of novel inhibitors of nonreplicating *Mycobacterium tuberculosis* using a carbon starvation model. Acs Chem Biol 8:2224-2234 doi:10.1021/cb4004817

Jakoby M, Ngouoto-Nkili CE, Burkovski A (1999) Construction and application of new *Corynebacterium glutamicum* vectors. Biotechnol Tech 13:437-441 doi:10.1023/A:1008968419217

Quandt J, Hynes MF (1993) Versatile suicide vectors which allow direct selection for gene replacement in gram-negative bacteria. Gene 127:15-21 doi:0378-1119(93)90611-6

Tauch A, Kirchner O, Löffler B, Götker S, Pühler A, Kalinowski J (2002) Efficient electrotransformation of *Corynebacterium diphtheriae* with a mini-replicon derived from the *Corynebacterium glutamicum* plasmid pGA1. Curr Microbiol 45:362-7 doi:10.1007/s00284-002-3728-3

Supplementary Table S2: Oligonucleotide primers used in the study.

S.No.	PCR Primers	Sequence (5' to 3')
1	p456F	CGTTGGCCGATTCATTATTGCAACAGCTGATTGCCCTTCACC
2	p456R	AGTGAGCTAACTCACATTATTGCGGCCGCGGATCAGCTTGCAATTC
3	VpA	GCTAGAGTCATATGTTCATGACTCCATTATTAG
4	VpB	CTCCTAGCTCTCGAGCGCAAAGAGAAAGCAG
5	REPpA	GAGTCATGAACATATGACTCTAGCCGATCCG
6	REPpB	CTCTTTGCGCTCGAGAGCTAGGAGCGAGACAC
7	VRNAA	AGGTATTTGTGCATAAAGCCTAAGGGGTAG
8	REPRNAA	TTAGGCTTTATGCACAAATACCTGAAAAAGTTG
9	Oppgen1F	GCCGGTACGTTCGCCATCTG
10	Oppgen1R	GTTGAGTTCATGCATGTCGTCGTCTCCGGGACTG
11	Nedgen1F	ACGACGACATGCATGAACTCAACGCCGGACTGAAC
12	Nedgen1R	GACGACGGGAAGCATGAAAC
13	CmF	TAGCCTAGGAGGTTGGGCGTCGCTTGGTC
14	CmR	TAGCCTAGGTCAGCTGTTGCCCGTCTCAC
15	AccD3F	CATATGACCACCACGACCGCAGAG
16	AccD3R	CGCGGTGGCGACCTACAG
17	UpperF	TTCGGCGAAGGAAGTCTG
18	UpperR	GGATGGTGAAGATGCCGACCTG
19	PMV10F	CCAATATTTCTTGCTTAGCTAATCGATAGCTGACGACGGGAAGCATGAAAC
20	PMV10R	CCTTTTTTTTTGGAGCTAGGTGATCGCCCTTGCCGGTACGTTC
21	RoCMF	CCTAGGTTCGCTGTGCGGGCTCTAAC
22	RoCMR	CCTAGGTCCTACGAGTTGCATGATAAAGAAG
23	UppaccD3F	ATGCATTTCGCCGACGCAACAGGTTCC
24	UppaccD3R	ACCGGTTCGCGGGTTCCTTTGCCGTTTC
25	dd112F	GCCCGAAATGAGCACGATCC
26	dd112R	CCTTCATCCGTTTCCACGGTAACGACGGCCAGTGCCAAGC
27	testGJ7firstrecombF3	AAACGACGGCCAGTGAATTG
28	testGJ7firstrecombR3	CCGACCTCGATGAAACTTCC
29	testGJ7firstrecombF2	CCGAGGAGTCGTACATGTTC
30	testGJ7firstrecombR2	GATGGCGTAGAGCTCACTGC
31	delaccD3F	CGGTGCCGACCTCACCAAGAAG
32	delaccD3R	TTGCCGTACTGGGCCTGGTTCC
33	Agl2sacBF	GGGTGTCTCGCTCCTAGCTCCACATATACCTGCCGTTCAC

34	Agl2sacBR	TACCTGCTTTCTCTTTGCGCTACGCGTCGACGGTATCGATAAG
35	testwt3F	GACGGCCGCAACCATTAC
36	testwt3R	CTTCCCACGTCAGTTCCATC
37	testmutF	GCAAGTTCAACGAGGCATTC
38	testmutR	AGGCCAGATTCTCACCATAG
39	testvecF2	CCAGCTCATCTGGCTCATTG
40	testvecR2	CATCGTCGCTAGAGCTTTCC

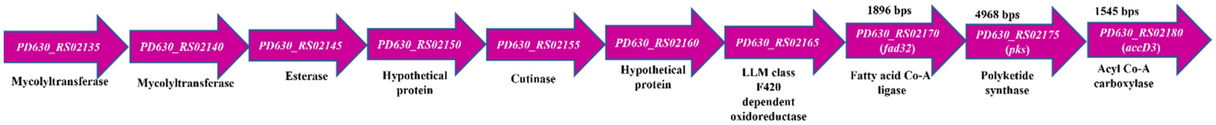

Fig. S1: Mycolic acid gene cluster in *R. opacus*. *fad32* and *accD3* are present upstream and downstream of *pks,* respectively in the *R. opacus* genome.

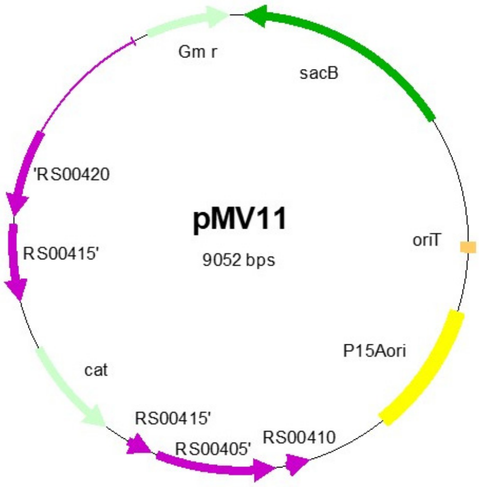

Fig. S2: Standard suicide recombination vector pMV11 based on pJQ200mp18. The elements shown are P15Aori for replication in *E. coli*, oriT for conjugative transfer, *cat* encodes resistance to chloramphenicol, Gm^r for gentamycin resistance and homologous arms flanking *cat.*

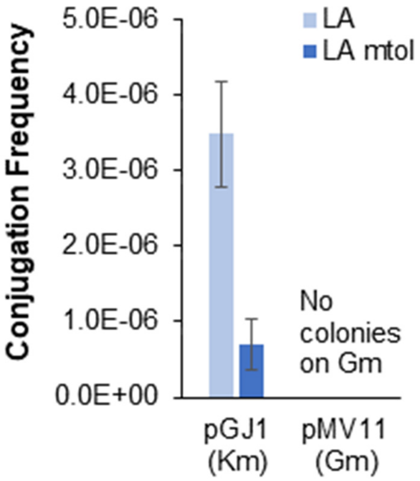

Fig. S3: Conjugation frequencies obtained using conditional suicide plasmid pGJ1 and standard suicide plasmid pMV11. Conjugation frequencies were defined as the number of colonies obtained on selective plates divided by the number of cells found on LA Nal. No colonies were obtained in the experiments using pMV11.

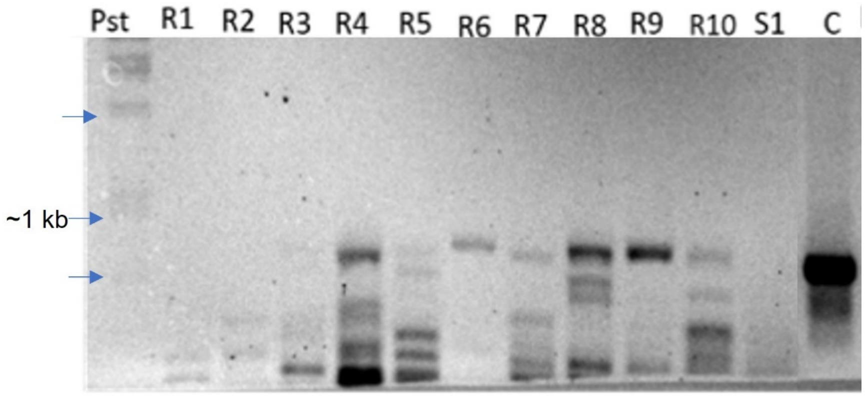

Fig S4: Homologous recombination with pGJ1. R1-R10 (Cm^r, Km^s, sucrose^r colonies), S1- Cm^s, Km^r sucrose^r colony, C-control (pGJ1) Primers- CmF/R, Expected band- 0.94 kb. R4, R6, R8 and R9 shows the expected band as control.

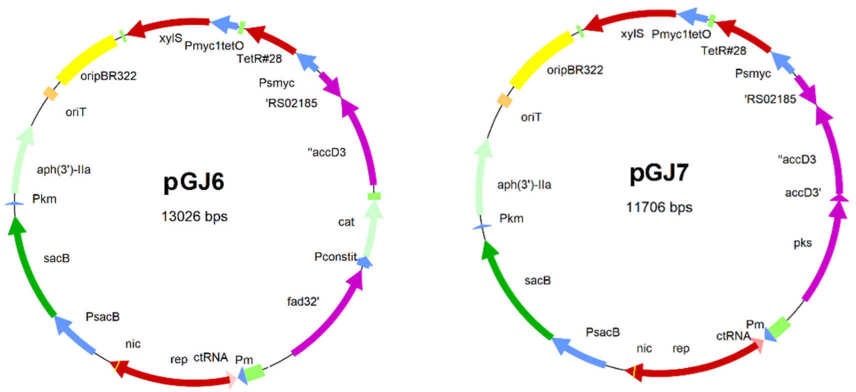

Fig. S5: Conjugative, conditional suicide plasmids pGJ6 and pGJ7 used to construct mycolic acid negative *R. opacus* mutants.

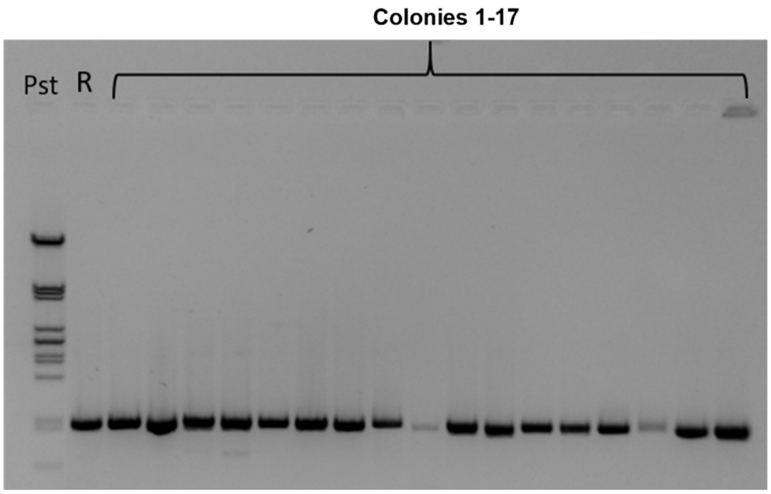

Fig. S6: Colony PCR to check *R. opacus*::pGJ6 mutant with primers testwt3F/R (1 kb). All Km^s colonies tested (1-17) after the second recombination showed the band indicative of the wild type strain. R: wild type *R. opacus* (Control)

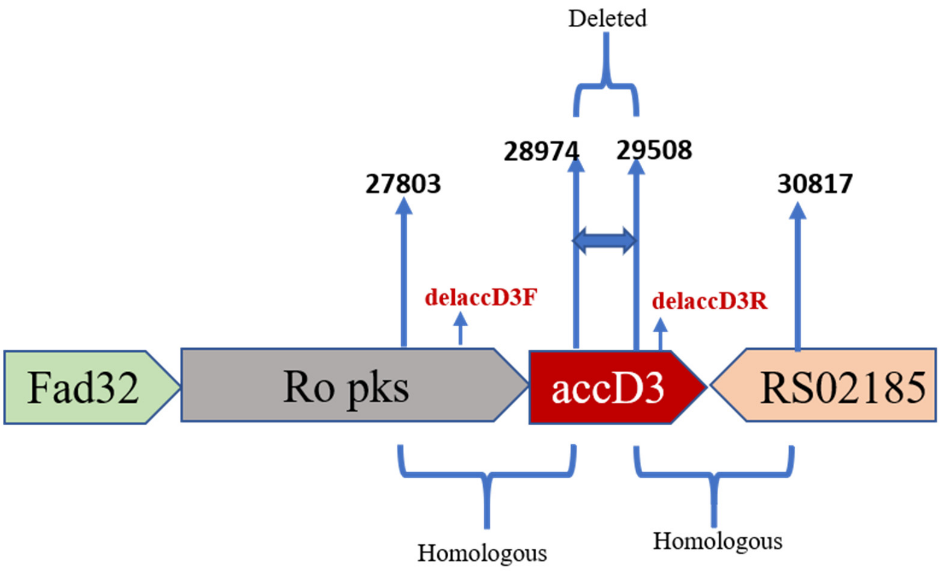

Fig. S7: Image showing part of the *accD3* partially deleted in plasmid pGJ7 and the location of primers used for testing putative mutants.

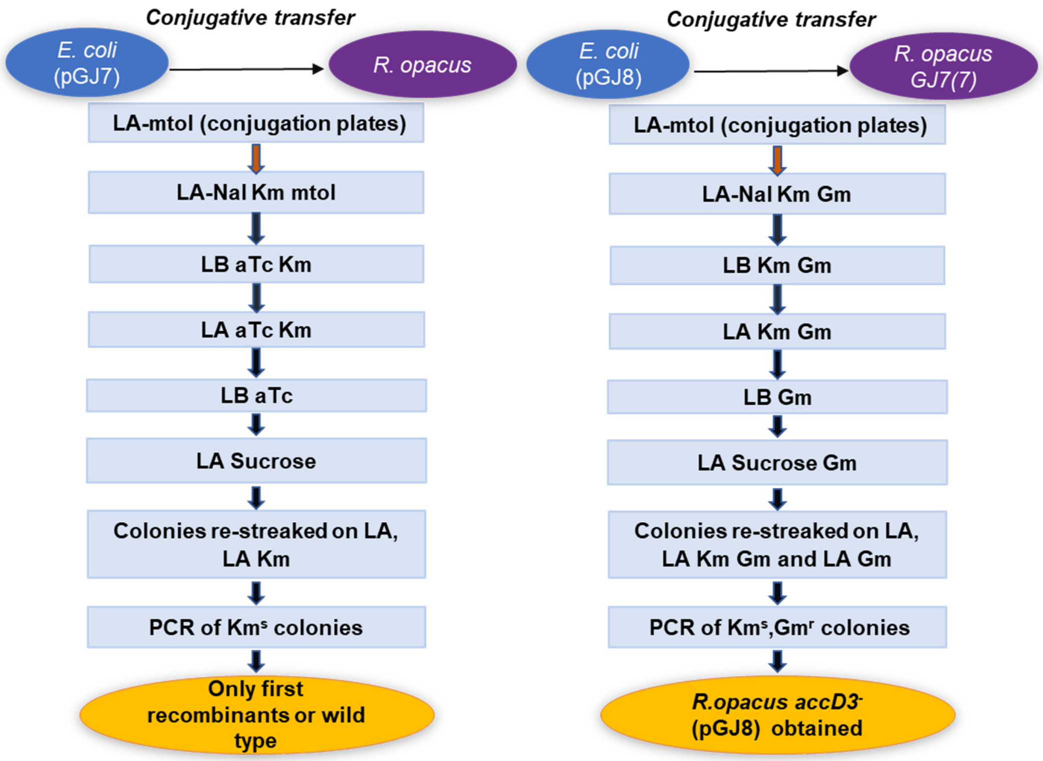

Fig. S8: Mutant construction steps for *R. opacus* Δ*accD3* mutant strain via homologous recombination

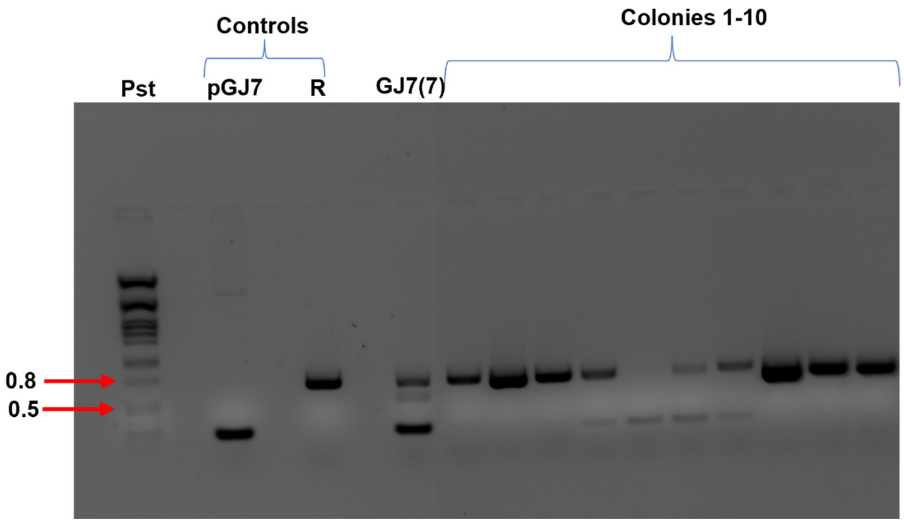

Fig. S9: PCR testing *R. opacus* Δ*accD3* mutant strain without complementation with primers delaccD3F/R. R: *R. opacus* wild type, GJ7(7): the strain selected after first recombination step. Colonies 1-10 were picked after sucrose selection. Expected bands for wild type: 0.719 kb, mutant: 0.192 kb and first recombinants: 12.4, 0.719 and 0.192 kb. All colonies (1-10) are either wild type or first recombinants.
